# Ranatensin-HL: A Bombesin-Related Tridecapeptide from the Skin Secretion of the Broad-Folded Frog, *Hylarana latouchii*

**DOI:** 10.3390/molecules22071110

**Published:** 2017-07-04

**Authors:** Yan Lin, Tianbao Chen, Mei Zhou, Lei Wang, Songkun Su, Chris Shaw

**Affiliations:** 1 College of Bee Science, Fujian Agriculture and Forestry University, Fuzhou 350002, China; 2 Natural Drug Discovery Group, School of Pharmacy, Queen’s University, Belfast BT9 7BL, Northern Ireland, UK; t.chen@qub.ac.uk (T.C.); m.zhou@qub.ac.uk (M.Z.); l.wang@qub.ac.uk (L.W.); chris.shaw@qub.ac.uk (C.S.)

**Keywords:** amphibian, peptides, mass spectrometry, molecular cloning, bombesin, smooth muscle

## Abstract

Bombesin-related peptides are a family of peptides whose prototype was discovered in amphibian skin and which exhibit a wide range of biological activities. Since the initial isolation of bombesin from *Bombina bombina* skin, diverse forms of bombesin-related peptides have been found in the skins across Anura. In this study, a novel bombesin-related peptide of the ranatensin subfamily, named ranatensin-HL, was structurally-characterised from the skin secretion of the broad-folded frog, *Hylarana latouchii*, through combination of molecular cloning and mass spectrometric methodologies. It is composed of 13 amino acid residues, pGlu-RAGNQWAIGHFM-NH_2_, and resembles an N-terminally extended form of *Xenopus* neuromedin B. Ranatensin-HL and its C-terminal decapeptide (ranatensin-HL-10) were chemically synthesised and subjected to in vitro smooth muscle assays in which they were found to display moderate stimulatory effects on rat urinary bladder and uterus smooth muscles with EC_50_ values in the range of 1–10 nM. The prepro-ranatensin-HL was highly homological to a bombesin-like peptide from *Rana catesbeiana* at both nucleotide and amino acid levels, which might provide a clue for the taxonomic classification of ranid frogs in the future.

## 1. Introduction

Amphibian skin has long been known as a delicate and multifunctional organ which has played a plethora of fundamental roles in their survival for millions of years. Such roles include respiration, camouflage, temperature control and chemical defence against encountered predators and pathogens [1]. The latter function is dependent on the secretions of highly specialised granular glands spread on the dorsal skin surface which contain a multitude of biologically-active peptides including antimicrobials, anticarcinogens, pheromones, neuropeptides and protease inhibitors [2,3]. One common class of peptides found in amphibian skin is the bombesin-related peptides, whose prototype is represented by bombesin, a tetradecapeptide originally isolated from the skin of *Bombina bombina* by Anastasi et al. as early as 1971 [4]. To date, a large number of bombesin-like peptides have been purified from the skin secretions of other anurans and these include ranatensin and phyllolitorin from *Rana* and *Phyllomedusa* species, respectively [1,5,6,7].

In 1979, the first mammalian homologue of amphibian bombesin was isolated from porcine stomach based on its capability of stimulating gastrin release from porcine antral tissue and was named gastrin-releasing peptide (GRP) in accordance with [8]. Neuromedin B (NMB) was the second mammalian counterpart of bombesin that was discovered, being isolated in 1983 from porcine spinal cord extracts based on its stimulation of rat uterus contractility [9]. Extensive studies on mammalian bombesin-like peptides have revealed that although multiple forms of GRP and NMB are distributed in many mammalian tissues, they are the unique homologues of bombesin-like peptides existing in mammals [10].

In accordance with phylogenetic analyses, bombesin-like peptides can be grouped into three branches—the GRP branch, the NMB branch and the bombesin-related peptides branch—which reflect the specific peptides originally characterised in amphibians consisting of bombesin, ranatensin and phyllolitorin subfamilies [11,12]. These subfamilies are differentiated by their pharmacological actions, receptor selectivity and amino acids near their carboxyl terminus: members of the bombesin subfamily have a C-terminus of -Gly-His-*Leu*-Met-NH_2_; the ranatensins are terminated with -Gly-His-*Phe*-Met-NH_2_; and the phyllolitorin group possess Gly-*Ser*-*Phe/Leu*-Met-NH_2_ at their C-terminals [1,13,14]. GRP and NMB can be classified into the bombesin and ranatensin subfamilies, respectively, due to their C-terminal structural homologies, yet the mammalian counterpart of phyllolitorins remain to be discovered, if they exist at all [10,15,16].

Amphibian skin bombesin-like peptides and their mammalian homologues have been shown to elicit various biological effects including direct action on extravascular smooth muscles, regulation of exocrine and endocrine secretion, autocrine tumour-growth effects, thermoregulation and mediation of satiety [11,17,18,19,20,21]. Such profound pharmacological effectiveness not only enables frogs to defend themselves against attacking predators like snakes, birds or mammals, but also provides scientists with many clues for understanding some aspects of disease pathogenesis and may assist in the diagnosis and/or therapy of some diseases.

Here, the identification, structural characterisation, molecular cloning of biosynthetic precursor cDNA and pharmacological investigation of a novel bombesin-related peptide, named ranatensin-HL, from the skin secretion of the broad-folded frog, *Hylarana latouchii*, are described. This peptide is a tridecapeptide which is essentially identical to *Xenopus* neuromedin B extended at its N-terminus by pGlu-Arg-Ala-. Both ranatensin-HL and its C-terminal decapeptide (ranatensin-HL-10), were synthesised and subjected to preliminary pharmacological analysis using rat urinary bladder and uterus smooth muscle preparations, indicating that they would evoke similar myotropic effects like most other bombesin-like peptides.

## 2. Results

### 2.1. Identification and Structural Characterisation of Ranatensin-HL

**Figure 1:**
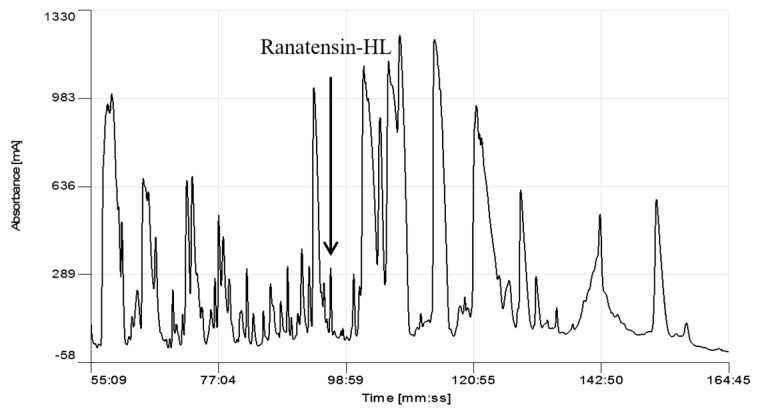
Region of reverse phase HPLC chromatogram of the skin secretion of *Hylarana latouchii*. The elution position/retention time of the pharmacologically-active peptide (ranatensin-HL), is indicated (arrow).

**Figure 2:**
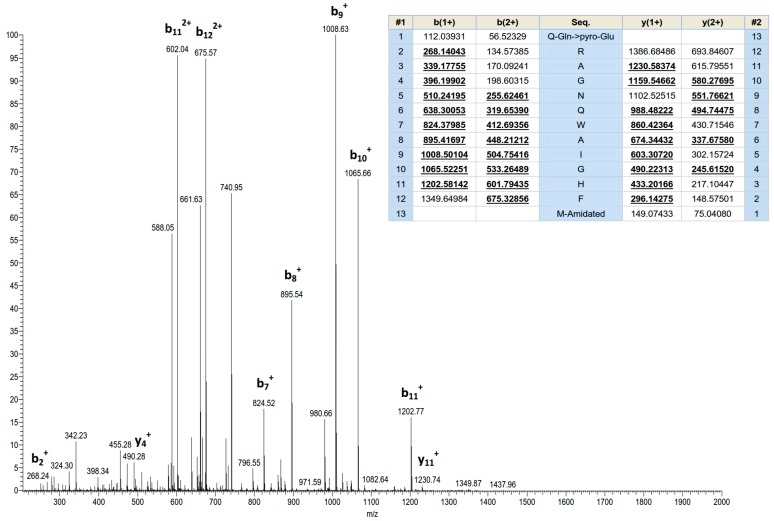
MS/MS fragmentation sequencing spectra of the bioactive peptide (ranatensin-HL) identified in the HPLC fraction of *Hylarana latouchii* skin secretion. Panel shows predicted singly and doubly charged *b*-ions and *y*-ions arising from MS/MS fragmentation. Observed ions are underlined and in bold typeface.

**Figure 3:**
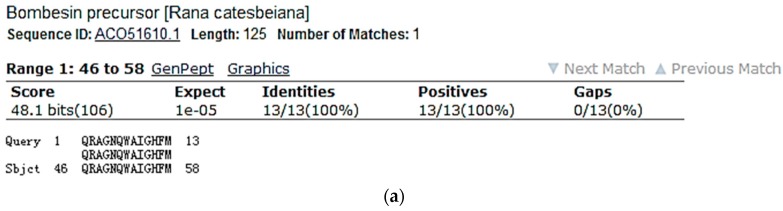

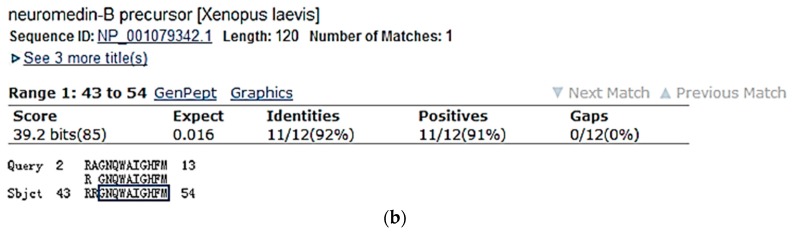
National Centre for Biotechnology Information-Basic Local Alignment Search Tool Protein Database (NCBI-BLASTp) analysis of the mature peptide amino acid sequence of ranatensin-HL. (**a**) Ranatensin-HL and bombesin precursor (*Rana catesbeiana*) amino acids 46–58 showing 100% identity. (Accession number: ACO51610.1); (**b**) Ranatensin-HL and neuromedin-B precursor (*Xenopus laevis*) amino acids 43–54 showing 92% identity (Accession number: NP 001079342.1); however, the C-terminal 10-amino acid of ranatensin-HL and the putative mature neuromedin-B peptide [22] showed 100% identity. The predicted mature peptide domain is boxed.

Screening of reverse phase HPLC fractions of *H. latouchii* skin secretion for pharmacological activity using rat urinary bladder and uterus smooth muscle preparations resulted in identification of a significant contractile activity in fraction # 94, coincident with a small but discrete absorbance peak as shown in Figure 1. Analysis of this fraction using matrix-assisted laser desorption/ionization time-of-flight (MALDI-TOF) mass spectrometry showed that it contained a single major peptide with mass of 1499.02 Da. The primary structure of this peptide was analysed by MS/MS fragmentation sequencing and subsequently confirmed by molecular cloning of the precursor-encoding cDNA to eliminate the L/I ambiguity (Figure 2). Bioinformatic analysis of the primary structure of the peptide using the National Centre for Biotechnology Information (NCBI) database indicated sequence identity with amino acids 46–58 of a bombesin precursor from *Rana catesbeiana* (Figure 3a) and the complete C-terminal 10-mer sequence was identical to a putative neuromedin-B peptide identified in the brain of *Xenopus laevis* (Figure 3b) [22]. Thus, this skin peptide was essentially an N-terminally extended form of *Xenopus* brain neuromedin B. As the C-terminal was -Gly-His-Phe-Met-NH_2_ coincident with ranatensin subfamily peptides, this bombesin-related peptide was considered as a member of ranatensin group and was named ranatensin-HL (HL = *Hylarana latouchii*) in accordance with a practical nomenclatural system proposed by Simmaco et al. [23].

### 2.2. Molecular Cloning of Ranatensin-HL Biosynthetic Precursor-Encoding cDNA

**Figure 4:**
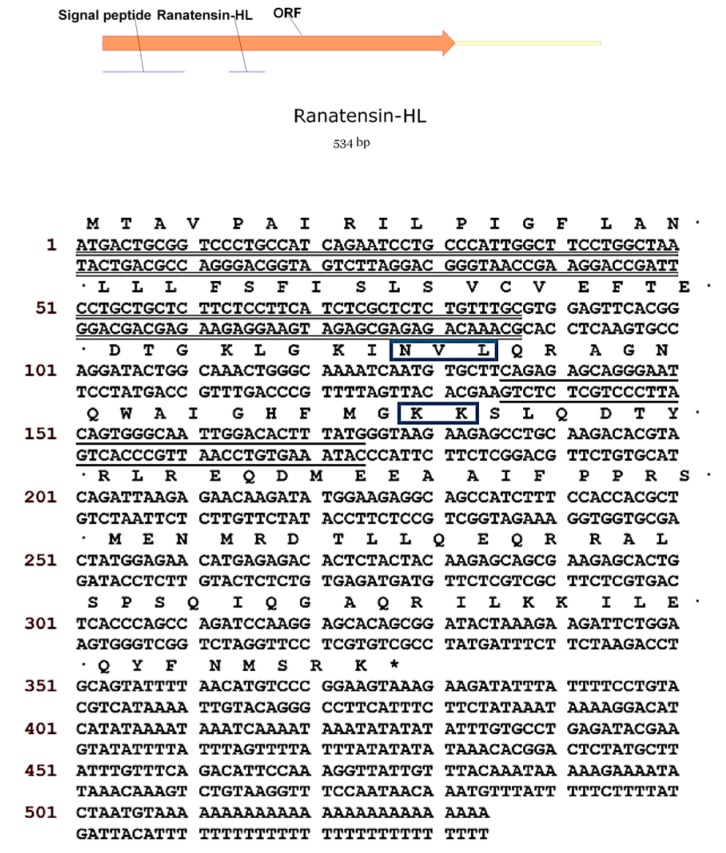
Nucleotide and open-reading frame translated amino acid sequences of cloned cDNA encoding the biosynthetic precursor of ranatensin-HL. The putative signal peptide is double-underlined, the mature peptide sequence is single-underlined and the stop codon is indicated by an asterisk. The proteolytic cleavage sites for the mature peptide are boxed.

**Figure 5:**
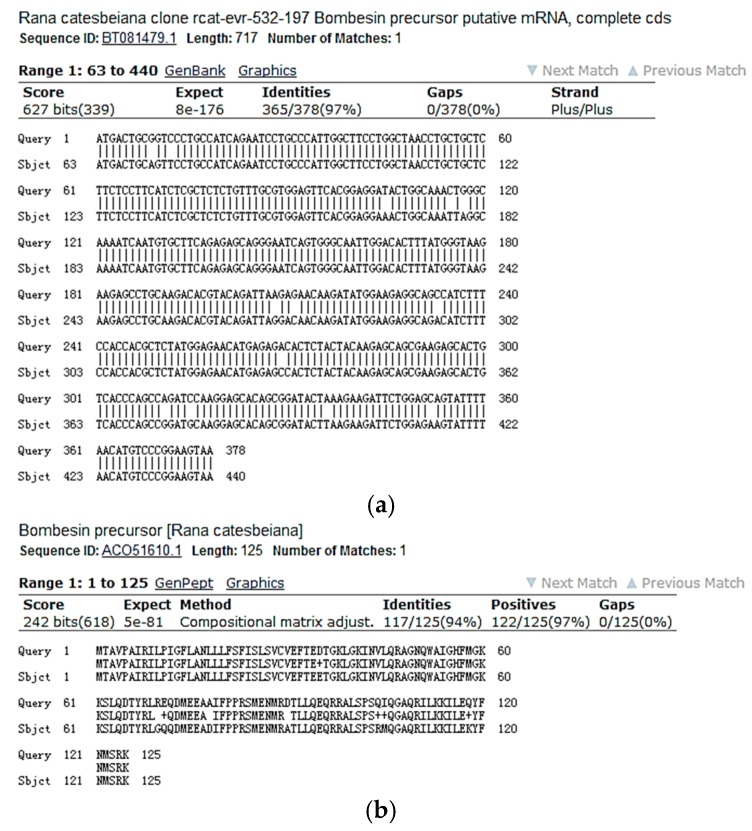
NCBI-BLAST analysis of the open-reading frame nucleotide and amino acid sequences of the ranatensin-HL biosynthetic precursor. (**a**) Ranatensin-HL ORF nucleotides 1–378 and bombesin precursor mRNA (*Rana catesbeiana*) nucleotides 63–440 showing 97% identity. (Accession number: BT081479.1); (**b**) Ranatensin-HL ORF amino acid 1–125 and bombesin precursor (*Rana catesbeiana*) amino acid 1–125 showing 94% identity. (Accession number: ACO51610.1).

Using the Rapid Amplification of cDNA Ends (RACE) PCR strategy described previously [24], the full-length cDNA encoding the biosynthetic precursor of ranatensin-HL was successfully and repeatedly cloned from the *H. latouchii* skin secretion-derived cDNA library. The open-reading frame (ORF) of the precursor protein was much longer than that of other amphibian ranatensin precursors, consisting of 125 amino acid residues. However, the domain architecture of this protein was similar to the precursors of other ranatensins, composed of a putative signal peptide, an N-terminal extension peptide followed by a putative propeptide convertase processing site (-NVL-), a single copy of mature peptide and a C-terminal extension peptide containing a further convertase processing site (-KK-) and a glycyl residue amide donor (Figure 4). Bioinformatic analysis of both the open-reading frame nucleotide and amino acid sequences of the ranatensin-HL precursor using National Centre for Biotechnology Information-Basic Local Alignment Search Tool Nucleotide Database (NCBI-BLASTn) and NCBI-BLASTp searches, revealed an extremely high degree of sequence identity with those of the bombesin precursor identified from *Rana catesbeiana* (Figure 5) (unpublished). The nucleotide sequence of the precursor-encoding cDNA of ranatensin-HL has been deposited in the European Molecular Biology Laboratory (EMBL) Nucleotide Sequence Database under the accession code LN626611.2.

### 2.3. Smooth Muscle Pharmacology

**Figure 6:**
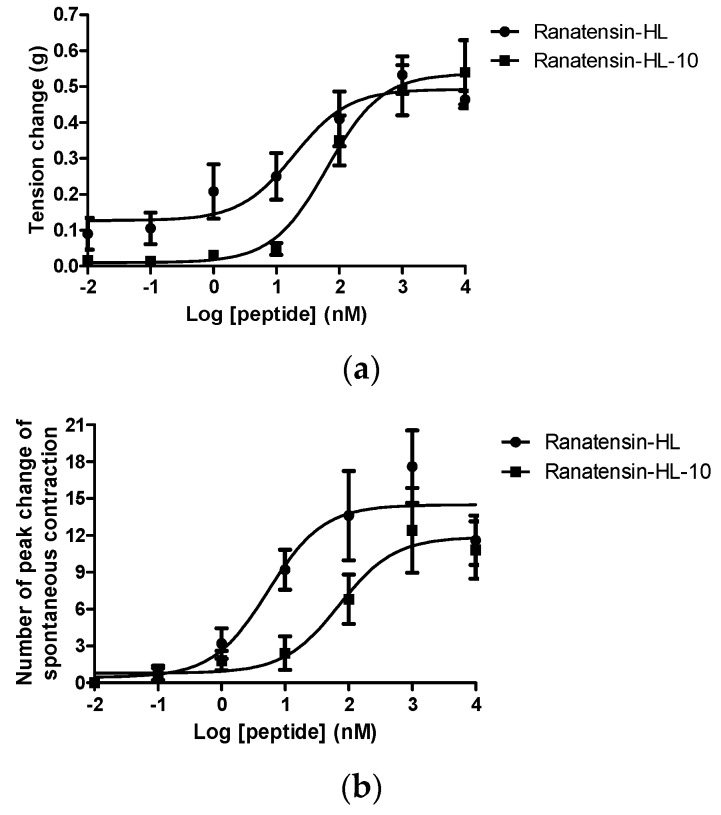
Comparison of myotropic effects of synthetic ranatensin-HL and ranatensin-HL-10 on isolated rat urinary bladder and uterus smooth muscles. (**a**) Dose-response curves of ranatensin-HL and ranatensin-HL-10 using rat urinary bladder smooth muscle preparations. EC_50_ values were determined as 19.2 and 63.8 nM, respectively; (**b**) Dose-response curves of ranatensin-HL and ranatensin-HL-10 using rat uterus smooth muscle preparations. EC_50_ values were determined as 5.4 and 70.9 nM, respectively. Each data point represents the mean and standard error of five determinations.

Both ranatensin-HL and ranatensin-HL-10 were successfully synthesised by solid-phase Fmoc methodology and were purified using reverse phase HPLC; the degree of purity and the structural authenticity were confirmed by MALDI-TOF mass spectrometry and MS/MS fragmentation analysis. The synthetic replicates were used to construct dose-response curves using rat urinary bladder and uterus smooth muscles. They were found to possess a moderate stimulation of contraction in rat urinary bladder smooth muscle and increased the frequency of spontaneous contraction of rat uterus smooth muscle in a dose-dependent manner (Figure 6). More specifically, ranatensin-HL and ranatensin-HL-10 exhibited similar contractive effects on rat urinary bladder smooth muscle with EC_50_ values of 19.2 and 63.8 nM, respectively; while on the other hand, ranatensin-HL was more potent on rat uterus smooth muscle with an EC_50_ (5.4 nM) nearly 15 times lower than that of ranatensin-HL-10 (70.9 nM).

## 3. Discussion

Amphibian skin is a remarkable source of bioactive peptides and it was predicted by Vittorio Erspamer (1909–1999) that each skin peptide would have a counterpart in mammalian tissues [25]. The majority of these amphibian skin peptides share highly conserved biologically active cores with their endogenous mammalian counterparts [25,26,27]. Additionally, in some cases, the peptides from the skin may have a wider distribution in other tissues of the amphibian. Bombesin-related peptides are a class of peptides appearing in the skin of many species of amphibians but also expressed in their central nervous systems (CNS) as well as their gastrointestinal (GI) tracts [12]. At the same time, they are found to have their homologues—GRP and NMB—in mammalian CNS and GI tract at considerably lower levels than those expressed in amphibian skin [15,17].

**Figure 7:**
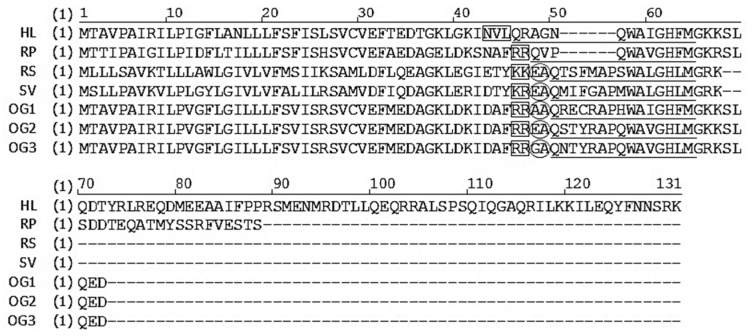
Sequence comparison of the precursors of bombesin-related peptides from different species of the Ranidae family. HL, *Hylarana latouchii* (this study); RP, *Rana pipiens*; RS, *Rana shuchinae*; SV, *Sanguirana varians*; OG1-OG3, *Odorrana grahami*. The sequences of mature peptide are underlined. The processing sites of the precursor for releasing mature peptides are boxed. The second possible processing sites are circled in some sequences. Gaps are introduced to optimise the sequence homology.

All bombesin-related peptides from amphibian skin are characterised by a conserved C-terminal region with the structure of -WAXGXXM-NH_2_, but differ widely in their N-terminal regions [28,29]. In the current study, a bombesin-related peptide of the ranatensin subfamily has been identified from the skin secretion of *Hylarana latouchii* and it terminates in the canonical C-terminal amidated tetrapeptide sequence—Gly-His-Phe-Met-NH_2_. It was named ranatensin-HL. As shown in Figure 4, the ranatensin-HL precursor contained a processing site (-KK-) that resembled those (-KK-, -KR-, -RK-) at the C-terminals of bombesin-related peptides from other amphibians, in which a dibasic amino acid cleavage site is present at the C-terminus. The glutamine and glycine residues, respectively, provided the N-terminal pyroglutamyl residue and the amide for the C-terminal methionine. The precursor sequence alignment of ranatensin-HL with homologues from other ranid frogs is illustrated in Figure 7 [13,28,29,30], which shows that the overall structure of the ranatensin-HL precursor is distinct from those of other ranid frog precursors. Compared with the average size (about 80 residues) of bombesin-related peptide precursors identified from members of the Ranidae family, ranatensin-HL was processed from a much larger precursor of 125 amino acid residues containing a 67-mer C-terminal extension peptide. Moreover, it was notable that unlike the majority of bombesin-related peptide precursors previously cloned from ranid frog skins whose N-terminals were usually flanked by the dibasic amino acid cleavage sites, Arg-Arg, Lys-Lys, Lys-Arg, Glu-Ala, Ala-Ala or Gly-Ala (Figure 7), ranatensin-HL was shown to be cleaved from its precursor following an Asn-Val-Leu sequence according to the structural analysis resulting from MS/MS fragmentation sequencing and this may represent a recognition sequence of a chymotryptic-like enzyme. Thus, the C-terminus of ranatensin-HL was released from the precursor by cleavage at dibasic amino acids; yet the N-terminus was released by a chymotryptic-like cleavage. In fact, the sizes and N-terminal processing patterns of bombesin-related peptide precursors differ significantly among different amphibian families but share similar properties within the same family, a factor which may be of possible use as a marker of taxonomic and molecular phylogenetics as suggested by Li et al. [30].

Through the bioinformatic analysis of the primary structure of ranatensin-HL performed using the NCBI-BLASTp search, it revealed that the C-terminal 10-amino acids of ranatensin-HL were identical to the predicted end-product from a gene in the brain of *Xenopus laevis*, whose product was named *Xenopus* neuromedin B [22] (Figure 3b). In this study, this C-terminal decapeptide was named temporarily as ranatensin-HL-10 due to its homology with ranatensin-HL. What was intriguing was whether there was a difference in terms of bioactivity between these two forms of peptides and, hence, we chemically synthesised both and evaluated their corresponding myotropic effects by conducting smooth muscle pharmacological assays. In keeping with other bombesin-related peptides, both ranatensin-HL and ranatensin-HL-10 displayed prompt and moderate contractile effects on rat urinary bladder and uterus smooth muscles, suggesting that they act on the same receptor.

As rantensin-HL and ranatensin-HL-10 possessed similar myoactivity, it prompted the thought that this alternative processing of ranatensin-HL to ranatensin-HL-10 might occur endogenously and that ranatensin-HL-10 might be present in the skin of *H. latouchii* and possibly that both may occur also in the frog’s brain. These questions would be interesting to address in future research. In the current study, ranatensin-HL-10 was not detected in the skin secretion of *H. latouchii* through either molecular cloning or bioactivity assessing of HPLC fractions; nevertheless, it could not exclude the probability of omission due to the trace amount present in the skin secretion and the detection limitation. Moreover, in the pharmacological bioassay, ranatensin-HL and ranatensin-HL-10 showed a similar potency on rat urinary bladder smooth muscle but differed in potency on rat uterus smooth muscle, indicating that the N-terminal region may contribute to myotropic potency to some extent. This result may likewise provide some clues for the future study of structure–function relationships of both peptides. Since bombesin receptors have a wide and specific distribution in mammals and display different affinities for different bombesin-related peptides, it would be meaningful to investigate the myoactivity of ranatensin-HL and ranatensin-HL-10 on other mammalian tissues, which may provide an opportunity for the discovery of a novel bombesin receptor and perhaps identify a ligand for the BBS-3 receptor.

An interesting finding in current study was the high degree of structural identity at both the nucleotide and amino acid levels, between the precursors of ranatensin-HL and a bombesin-like peptide found in *Rana catesbeiana* (Figure 5). As a matter of fact, *Hylarana latouchii* is a member of the genus *Hylarana* within the Ranidae family and is endemic to central and southern China, while *Rana catesbeiana* belongs to the genus *Rana* (now *Lithobates*) of the Ranidae family and is native to North America. Taking the high degrees of structural similarities of precursor sequences into consideration, even though these two species have significant zoogeographical isolation, it seems that either one of these genes has evolved from the other or both have evolved from a common ancestor. This could come about if both share a close evolutionary strategy which may result from adaption to a similar ecological niche. Since molecular aspects have been employed more frequently recently for taxonomic purposes, the current data would provide an additional insight into the taxonomic classification of ranid frogs and perhaps a more systematic approach to generic assignations within the Ranidae family [31,32].

## 4. Materials and Methods

### 4.1. Specimen Biodata and Secretion Harvesting

Specimens of *Hylarana latouchii* were captured in Fujian Province, P.R. China. All frogs were adults and skin secretion was harvested by mild electrical stimulation on the dorsal skin surface of the frogs according to the methods described previously [33]. The obvious white mucous secretion was washed off using deionised water, collected, snap-frozen in liquid nitrogen, lyophilised and stored at −20 °C prior to use.

### 4.2. Reverse-Phase HPLC Fractionation of Skin Secretion

Five milligrams of lyophilised *H. latouchii* skin secretion was dissolved in 1 mL of trifluoroacetic acid (TFA)/water (0.05/99.95, *v/v*) and clarified by centrifugation. The clear supernatant was decanted and subjected to reverse-phase HPLC fractionation using a Cecil Adept CE4200 HPLC system (Amersham Biosciences, Buckinghamshire, UK) fitted with a Jupiter C-5 semi preparative column (300 Å, 5 µm, 25 cm × 1 cm, Phenomenex, Macclesfield, Cheshire, UK) and a Powerstream HPLC software, being eluted with a linear gradient formed from 0.05/99.95 (*v/v*) TFA/water to 0.05/19.95/80.00 (*v/v/v*) TFA/water/acetonitrile over 240 min at a flow rate of 1 mL/min. Fractions (1 mL) were collected automatically at 1 min intervals and the effluent absorbance was monitored at λ214 nm. Samples (100 μL) were removed from each chromatographic fraction, lyophilised and stored at −20 °C prior to analysis of myoactivity using rat urinary bladder and uterus smooth muscle bioassays.

### 4.3. Bioactivity Screening Using Rat Urinary Bladder and Uterus Smooth Muscles

Female Wister rats (250–300 g) were euthanized by carbon dioxide asphyxiation followed by cervical dislocation in accordance with institutional animal experimentation ethics and UK animal research guidelines. Procedures had been vetted by the Institutional Animal Care and Use Committee (IACUC) of Queen’s University Belfast. The rats were laid on their dorsal surface, followed by incising along the mid ventral line to open the abdomen and dissecting the subcutaneous fat. The exposed urinary bladder and intact uterine horns were removed from each rat and placed in ice-cold Kreb’s solution (118 mM NaCl, 4.7 mM KCl, 25 mM NaHCO_3_, 1.15 mM NaH_2_PO_4_, 2.5 mM CaCl_2_, 1.1 mM MgCl_2_ and 5.6 mM glucose) which was vigorously aerated with 95% O_2_ and 5% CO_2_. For urinary bladder smooth muscle preparations, muscle strips (2 mm × 10 mm) were dissected from the bladder under a dissection microscope and were pierced at each end by triangular hooks (0.2 mm diameter) which were tied with fine silk ligatures; for uterus smooth muscle preparations, each uterine horn was halved and 2 triangular hooks threaded the lumen. Then, the tissues were mounted on a transducer prior to placing in 2 mL organ baths containing Kreb’s solution at 37 °C following at 2 mL/min with constant bubbling of 95% O_2_ and 5% CO_2_. The tissue preparations were equilibrated for 1 h before experimental procedures were initiated. Samples of sequential reverse phase HPLC fractions were reconstituted in 22 μL of Kreb’s solution and used to screen for smooth muscle activity.

### 4.4. Identification and Structural Characterisation of Peptide Possessing Bioactivity

The reverse-phase HPLC fraction which was found to possess contractile activity on rat urinary bladder and rat uterus smooth muscles was initially analysed using MALDI-TOF MS (Voyager DE, PerSeptive Biosystems, Foster City, CA, USA) to determine the molecular mass. The major peptide present in the HPLC fraction was subjected to primary structural analysis by MS/MS fragmentation sequencing using an LCQ-Fleet electrospray ion-trap mass spectrometer (Thermo Fisher Scientific, San Francisco, CA, USA).

### 4.5. Molecular Cloning of the Peptide Biosynthetic Precursor-Encoding cDNA

A 5 mg sample of lyophilised skin secretion was dissolved in 1 mL of cell lysis/mRNA protection buffer supplied by Dynal Biotec, Wirral, UK. Polyadenylated mRNA was isolated using magnetic oligo-dT beads as described by the manufacturer (Dynal Biotech, Merseyside, UK), and was subsequently reverse-transcribed. The resultant cDNA library was subjected to 5′ and 3′-RACE procedures to obtain full-length prepro-peptide nucleic acid sequence data using a SMART-RACE kit (Clontech, Palo Alto, CA, USA) essentially as described by the manufacturer. Briefly, the 3′-RACE reactions employed a Nested Universal Primer (NUP) primer (supplied with the kit) and a degenerate sense primer (S: 5′-ARMGIGCIGGIAAYCARTGGGC-3′) that was complementary to the N-terminal amino acid sequence, pGlu-RAGNQWA-, of the bioactive peptide. PCR products were gel-purified and cloned using a pGEM-T vector system (Promega Corporation, Southampton, UK) and sequenced using an ABI 3100 automated sequencer (Applied Biosystems, Foster City, CA, USA). The sequence data obtained from the 3′-RACE product was used to design a specific antisense primer (AS: 5′-CGTATCTCAGGCACAAATATATA-3′) to a defined conserved site within the 3′ non-translated region of the peptide encoding transcript. 5′-RACE reaction was carried out using this primer in conjunction with the NUP primer and generated PCR products were gel-purified, cloned and sequenced as described above.

### 4.6. Solid-Phase Peptide Synthesis of Ranatensin-HL and Ranatensin-HL-10

Once the unequivocal primary structure of the bombesin-like peptide (ranatensin-HL) had been established through MS/MS fragmentation sequencing and translating cloned cDNA, it was synthesised by solid-phase Fmoc chemistry using a PS3 automated solid-phase peptide synthesiser (Protein Technologies, Tucson, AZ, USA). Products were purified by reverse-phase HPLC, and the degree of purity and authenticity of structure were confirmed by MALDI-TOF MS and MS/MS fragmentation sequencing. In addition, the C-terminal decapeptide of ranatensin-HL (ranatensin-HL-10) was also synthesised and purified in the same way.

### 4.7. Pharmacological Assay of Synthetic Peptides Using the Rat Urinary Bladder and Uterus Smooth Muscles

Solutions of synthetic peptides, ranging in concentration from 10^−2^ to 10^4^ nM, were made in Kreb’s solution and were used to construct dose-response curves. They were added to the bladder and uterus muscle strips which were put under 0.5 g of tension, in increasing concentrations with 10 min washes and 10 min equilibration periods between each dose. Each concentration of peptides was applied to a minimum of five muscle strips. Changes in tension of the bladder muscle strips were recorded and amplified through pressure transducers connected to a PowerLab System (AD Instruments Pty Ltd., Oxford, UK), while changes in spontaneous contraction frequency of the uterus smooth muscle preparations were recorded instead. Data were analysed to obtain the mean and standard error of responses by Student’s *t*-test and dose-response curves were constructed using a “best-fit” algorithm through the data analysis package provided. Responses were plotted as changes in grams of tension (for urinary bladder) or changes in spontaneous contraction frequency (for uterus) against final molar concentration of peptides present in the organ baths.

## 5. Conclusions

In this study, a bombesin-related tridecapeptide, ranatensin-HL (pGlu-RAGNQWAIGHFM-NH_2_), from the skin secretion of *Hylarana latouchii*, was structurally and pharmacologically-characterised. The precursor of ranatensin-HL was found to be highly identical to that of a bombesin-like peptide from *Rana catesbeiana* at both nucleotide and amino acid levels. The complete C-terminal 10-amino acids of mature ranatensin-HL (ranatensin-HL-10) was the same as *Xenopus* brain neuromedin B, thus ranatensin-HL was considered as the N-terminally extended form of neuromedin B. In the smooth muscle pharmacological assay, both ranatensin-HL and ranatensin-HL-10 exhibited moderate stimulation of contraction in rat urinary bladder and uterus smooth muscles.

## Abbreviations

The following abbreviations were used in this manuscript:

HPLC	High-Performance Liquid Chromatography
NCBI-BLASTp	National Centre for Biotechnology Information-Basic Local Alignment Search Tool Protein Database
NCBI-BLASTn	National Centre for Biotechnology Information-Basic Local Alignment Search Tool Nucleotide Database
RACE	Rapid Amplification of cDNA Ends
ORF	Open-Reading Frame
EMBL	European Molecular Biology Laboratory
Fmoc	9-Fluorenylmethyloxycarbonyl
TFA	Trifluoroacetic Acid
IACUC	Institutional Animal Care and Use Committee
MALDI-TOF MS	Matrix-Assisted Laser Desorption/Ionization Time-of-Flight Mass Spectrometry
NUP	Nested Universal Primer
